# A novel autism-associated *KCNB1* mutation dramatically slows Kv2.1 potassium channel activation, deactivation and inactivation

**DOI:** 10.3389/fncel.2024.1438101

**Published:** 2024-07-29

**Authors:** Rían W. Manville, Samantha D. Block, Claire L. Illeck, Jessica Kottmeier, Richard Sidlow, Geoffrey W. Abbott

**Affiliations:** ^1^Bioelectricity Laboratory, Department of Physiology and Biophysics, School of Medicine, University of California, Irvine, CA, United States; ^2^Faculty of Health Sciences, Ben-Gurion University of the Negev, Be’er Sheva, Israel; ^3^Medical School for International Health, Ben-Gurion University of the Negev, Be’er Sheva, Israel; ^4^Department of Pediatric Genetics, Children’s Hospital, University of Missouri, Columbia, MO, United States

**Keywords:** autism, absence seizures, developmental delay, *KCNB1*, Kv2.1

## Abstract

*KCNB1*, on human chromosome 20q13.3, encodes the alpha subunit of the Kv2.1 voltage gated potassium channel. Kv2.1 is ubiquitously expressed throughout the brain and is critical in controlling neuronal excitability, including in the hippocampus and pyramidal neurons. Human *KCNB1* mutations are known to cause global development delay or plateauing, epilepsy, and behavioral disorders. Here, we report a sibling pair with developmental delay, absence seizures, autism spectrum disorder, hypotonia, and dysmorphic features. Whole exome sequencing revealed a heterozygous variant of uncertain significance (c. 342 C>A), p. (S114R) in *KCNB1*, encoding a serine to arginine substitution (S114R) in the N-terminal cytoplasmic region of Kv2.1. The siblings’ father demonstrated autistic features and was determined to be an obligate *KCNB1* c. 342 C>A carrier based on familial genetic testing results. Functional investigation of Kv2.1-S114R using cellular electrophysiology revealed slowing of channel activation, deactivation, and inactivation, resulting in increased net current after longer membrane depolarizations. To our knowledge, this is the first study of its kind that compares the presentation of siblings each with a *KCNB1* disorder. Our study demonstrates that Kv2.1-S114R has profound cellular and phenotypic consequences. Understanding the mechanisms underlying *KCNB1*-linked disorders aids clinicians in diagnosis and treatment and provides potential therapeutic avenues to pursue.

## Introduction

Developmental encephalopathies constitute a heterogenous group of neurodevelopmental disorders (Bar et al., 2020a,b). Most patients with developmental encephalopathies are diagnosed during early childhood. Symptoms persist throughout life, and can include social, cognitive, motor, language, and behavioral impairments. The concept of “developmental and epileptic encephalopathy” (DEE) refers to the frequently associated epileptiform activity that can contribute to developmental plateauing or regression (Scheffer et al., 2017). Recent studies have highlighted the role of ion channels in the pathogenesis of DEEs (Wang et al., 2017; Raga et al., 2021).

The *KCNB1* gene encodes the alpha subunit of the Kv2.1 voltage gated potassium channel on chromosome 20q13.3. Voltage-gated potassium (Kv) channels form the largest family of ion channels in the human genome and are ubiquitously expressed throughout the human body. Kv channels are critical for regulating various excitable and non-excitable physiological processes, including skeletal and cardiac muscle contraction, nervous signaling, neurotransmitter and hormone release, and cell proliferation (Abbott, 2020).

**Figure 1:**
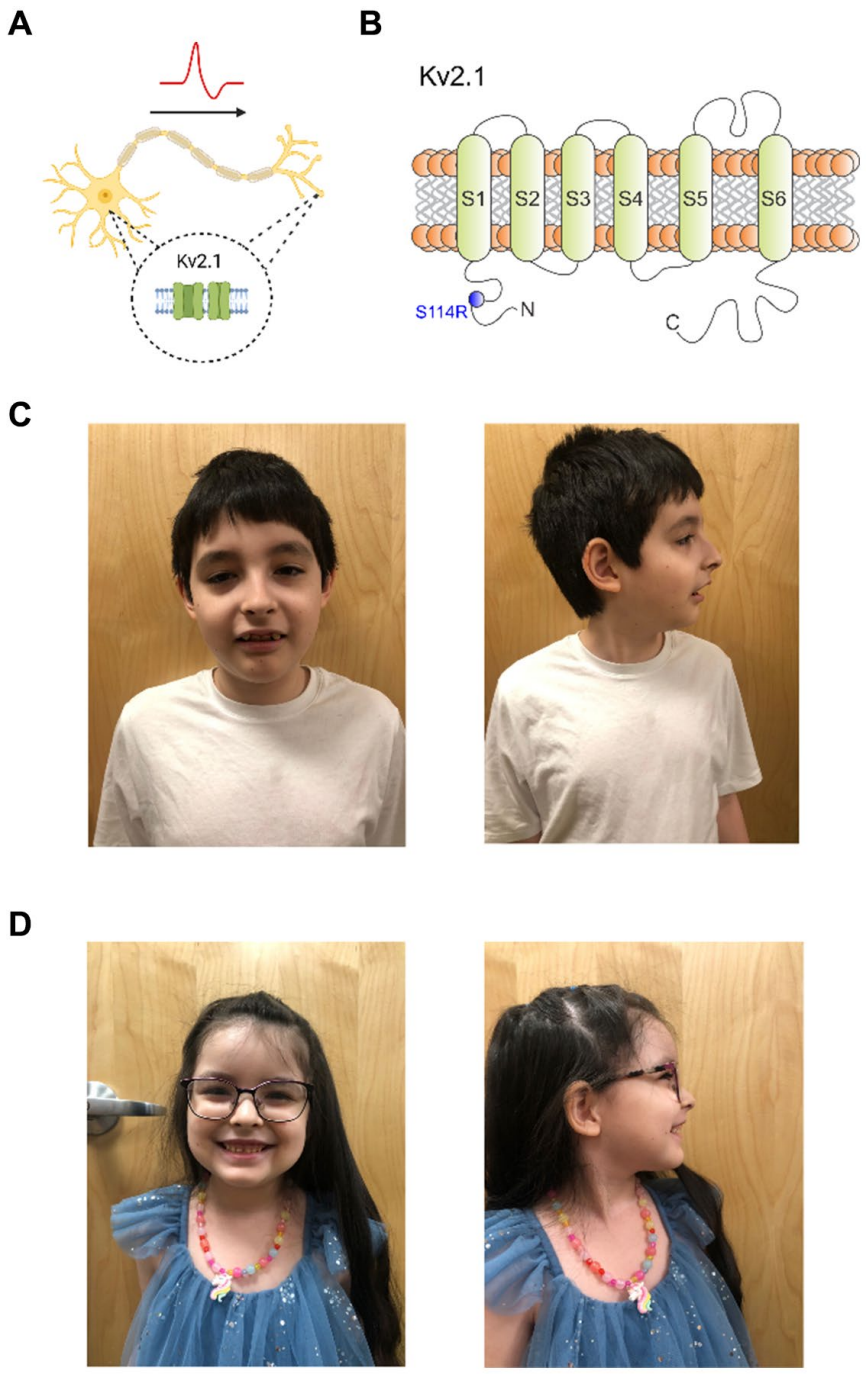
Kv2.1-S114R is associated with a multifaceted *KCNB1* encephalopathy. **(A)** Cartoon depicting the expression of Kv2.1 channel in the soma and dendrites of neurons. **(B)** Cartoon depicting the topology of Kv2.1 channels, the location of previously characterized mutations discovered in epilepsy and neurodevelopmental disorder patients, and the location of S114 in the N-terminal domain. **(C)** Frontal and sagittal photos of patient A demonstrate an oblong-shaped face, wide eyebrows with lateral thinning, moderately thin and downsloping palpebral fissures with mild hypertelorism, wide nasal tip, low and wide columella, slight micrognathia, and wide malaligned teeth. **(D)** Frontal and sagittal photos of patient B demonstrate prominent forehead with mild bossing, mild hypertelorism, wide and fleshy nose, low and wide columella, widely spaced large teeth.

Kv2.1, a delayed rectifier Kv channel, is expressed widely throughout the brain (Bishop et al., 2015; Bar et al., 2020a; Veale et al., 2022) and is required for neuronal membrane repolarization (Ottschytsch et al., 2002; de Kovel et al., 2017). Despite its ubiquity, Kv2.1 expression in neurons at the subcellular level is highly specific to the soma, proximal dendrites, and axon initial segment (AIS) (Figure 1A) (Trimmer, 1991; Scannevin et al., 1996). Thus, Kv2.1 plays a critical role in controlling neuronal excitability, including regulating somatodendritic excitability in hippocampal and pyramidal neurons (Bekkers, 2000; Du et al., 2000; Korngreen and Sakmann, 2000).

Heterozygous, pathogenic variants in the *KCNB1* gene can contribute to a diverse phenotype of neurodevelopmental disorders, ranging from DEEs to global development delay with or without epileptic activity (Bar et al., 2020a,b). If present, epileptiform activity typically occurs during infancy or childhood, and is often unresponsive to antiepileptic treatment. Features of DEEs can include photosensitivity, sleep activation abnormalities on EEG, language and speech difficulties, behavioral problems, hypotonia, spasticity, and ataxia. Abnormalities in magnetic resonance imaging (MRI), including atrophy and nonspecific periventricular white matter abnormalities, have been described in some individuals (de Kovel et al., 2017; Bar et al., 2020a,b).

To date, 55 *KCNB1* mutations have been reported in patients with encephalopathic epilepsy, infantile epilepsy, autism, and neurodevelopment disorders, and are located throughout Kv2.1 (Figure 1B) (de Kovel et al., 2017; Bar et al., 2020a; Xiong et al., 2022). However, only two mutations have been previously discovered in the N-terminal cytoplasmic region, P17T and E43G (Bar et al., 2020a; Veale et al., 2022). Here, we report a *KCNB1* sequence variant encoding a substitution in the N-terminus of Kv2.1, which we discovered in a male/female sibling pair with neurological disorders including autism, absence seizures and developmental delay. Functional characterization revealed an unexpected and complex perturbation of function in the mutant channel.

## Methods

### Human genome sequencing

We received a signed case report consent form from the legal guardian of the children. Both siblings had whole exome sequencing performed at GeneDx (Gaithersburg, MD, United States) using paired-end reads on an Illumina platform. Sequence reads were aligned to human genome build GRcH37/USCS hg19. Data were filtered using GeneDx’s custom analysis tool (XomeAnalyzer). The variant was reported as a variant of uncertain significance in accordance with the American College of Medical Genetics and Genomics (ACMG) criteria based on transcript NM_004975.2 (Richards et al., 2015).

### Preparation of channel subunit cRNA and *Xenopus laevis* oocyte injection

cDNA encoding human *KCNB1* was sub-cloned into a *Xenopus* expression vector (pMAX) incorporating *Xenopus laevis* β-globin 5′ and 3’ UTRs flanking the coding region to enhance translation and cRNA stability by Genscript (Piscataway, NJ, United States). The mutant *KCNB1* construct was generated by Genscript and subcloned into pMAX as above. cRNA transcripts were generated by *in vitro* transcription using the T7 mMessage mMachine kit (Thermo Fisher Scientific, Waltham, MA, United States) according to manufacturer’s instructions, after vector linearization with PacI. Stage V and VI defolliculated *Xenopus laevis* oocytes (Xenoocyte, Dexter, MI, United States) were injected with the channel cRNAs (2 ng) and incubated at 16°C in Barth’s solution containing penicillin and streptomycin, with daily washing, prior to two-electrode voltage-clamp (TEVC) recording.

### Two-electrode voltage clamp

TEVC was conducted at room temperature with an OC-725C amplifier (Warner Instruments, Hamden, CT, United States) and pClamp10 software (Molecular Devices, Sunnyvale, CA, United States) 24 h after cRNA injection. Oocytes, in a small-volume oocyte bath (Warner), were viewed with a dissection microscope for cellular electrophysiology. Extracellular bath solution (in mM): 96 NaCl, 4 KCl, 1 MgCl_2_, 0.3 CaCl_2_, and 10 HEPES, adjusted to pH 7.6 with TRIS BASE. Solutions were introduced into the oocyte recording bath by gravity perfusion at a constant flow of 1 mL per minute. Pipettes (1–2 MΩ resistance) were filled with 3 M KCl. Current–voltage graphs were measured in response to voltage pulses between −80 mV and +40 mV at 10 mV intervals from a holding potential of −80 mV. Conductance graphs were measured from tail currents generated at −40 mV immediately following the prepulse and normalized to the maximal current. Conductance was plotted as a function of voltage and fitted with a single Boltzmann function:


(1) $$g=\frac{\left(A_{1}-A_{2}\right)}{\left\{1+\exp\;\left[V_{\frac{1}{2}}-V/V_{s}\right]\right\}\;y+A_{2}}$$

where *g* is the normalized tail conductance, *A*_1_ is the initial value at −∞, *A*_2_ is the final value at +∞, *V*_1/2_ is the half-maximal voltage of activation and *V*_s_ the slope factor. We fitted activation and deactivation kinetics with single exponential functions.

#### Activation and deactivation kinetics

Activation kinetics were measured in response to voltage pulses between −10 mV and +40 mV at 10 mV intervals from a holding potential of −80 mV. Deactivation kinetics were measured between −120 mV and −60 mV in 10 mV intervals immediately following a +40 mV prepulse from a holding potential of −80 mV. Activation and deactivation traces were each fitted with a single exponential function.

#### Inactivation and recovery from inactivation

Inactivation was measured in response to a single voltage pulse at +40 mV for 10 s and 20 s from a holding potential of −80 mV. The percentage of inactivation was derived from the difference between the peak and plateau of the current at +40 mV. Fraction of non-inactivated channels was measured in response to 10 s voltage pulses between −80 mV and +10 mV immediately prior to a +40 mV voltage pulse from a holding potential of −80 mV. Fraction of non-inactivated channels was measured from the +40 mV voltage pulse, normalized to the maximal current, and fitted with a single Boltzmann function (Eq. 1). Recovery from inactivation was measured in response to consecutive 5 s +40 mV pulses at interpulse intervals of increasing duration from 0.01 to 30 s. The subsequent peaks of these pulses were then divided against the initial pulse and plotted as a function of the interpulse interval.

All data were analyzed using Clampfit (Molecular Devices) and Graphpad Prism software (GraphPad, San Diego, CA, United States).

### Statistics and reproducibility

All values are expressed as mean ± SEM. At least 2 batches of oocytes were used per experiment. Multiple comparison statistics were conducted using a One-way ANOVA with a Dunnett’s test for multiple comparisons. Comparison of two groups was conducted using a *t*-test; all *p*-values were two-sided. All electrophysiological data and statistics are summarized in the Supplementary material.

## Results

### Clinical phenotype

The sibling pair consisted of a 10-year-old boy (patient A) and a 6-year-old girl (patient B). Family history was significant for learning disability, dyslexia, autistic features and red-green color blindness in their father, and developmental delays and major depressive disorder in their mother. Their maternal grandmother had learning disability, syncopal episodes and severe muscle weakness. The parents are non-consanguineous.

Patient A was born after an uncomplicated pregnancy at 41 weeks gestation to a 23-year-old G1P1 mother via C-section for prolonged and worsening decelerations. His mother denied any drug, cigarette, or alcohol use during pregnancy nor was any genetic testing performed. His birthweight was 3.015 kg (19th percentile) and head circumference was 35.50 cm (43rd percentile). His Apgar scores were 2, 5, 6, 8 at 1, 5, 10 and 15 min, respectively, due to initially being limp, apneic, and bradycardic requiring a fluid bolus, positive pressure ventilation and CPAP for 30 min immediately postpartum. He was transferred to the neonatal intensive care unit for concerns of hypoxic ischemic encephalopathy and respiratory distress. At 4 days of age there was concern about the infant’s swallowing capabilities as he was having difficulty breathing and feeding via nasogastric tube simultaneously. No abnormalities were noted on a swallowing study, and brain MRI at 5 days of life was also unremarkable. He was discharged from the NICU on normal feeds at 14 days of life having passed newborn hearing screening and subsequent newborn metabolic screening also being normal.

From age 1–2, he demonstrated delayed achievement of developmental milestones, walking at 24 months and speaking at 36 months. Physical, occupational, and speech therapy were initiated. Multiple physical examinations by various providers over the next few years revealed jerky, poorly controlled upper extremity movements and ataxic gait leading to a diagnosis of cerebral palsy.

Brain magnetic resonance imaging (MRI) at 7 years of age again revealed no abnormalities and an EEG performed at the same time showed mild encephalopathy. At 3 years of age, the patient began demonstrating repetitive speech and behaviors along with limited social skills. He was diagnosed with autism spectrum disorder.

A hearing test along with urine organic acids/plasma amino acids were all normal. A Comprehensive Neuromuscular Disorders Panel (Invitae) covering 211 genes was ordered. This panel revealed a heterozygous VUS in HNRNPDL c.259 C>T/p.Arg87Cys causative of autosomal dominant limb-girdle muscular dystrophy type 1G (LGMD1G), a phenotype inconsistent with patient A’s presentation with a gnomAD frequency of 0.007%, *in silico* ambiguity as to the deleterious nature of this variant on protein structure/function, and no reports of this variant being causative of LGMD1G in the literature.

At 10 years of age a genetic evaluation was performed for the combined complaints of DD/ID, ASD, absence seizures, hypotonia, muscle cramping (upper extremity >lower extremity), anxiety, sleep disturbances and visual hallucinations. Ophthalmologic examination revealed myopia, astigmatism and red-color deficiency in both eyes. There was no history of developmental regression, persistent vomiting, rhabdomyolysis, or unexplained coma but perhaps lethargy post carbohydrate or protein intake. Morphometrics were normal including head circumference. Physical examination revealed an oblong-shaped face, wide eyebrows with lateral thinning, moderately thin and downsloping palpebral fissures with mild hypertelorism, wide nasal tip, low and wide columella, slight micrognathia, wide misaligned teeth (Figure 1C), and a solitary café-au-lait macule on the lower right leg. Neurologic examination was normal except he was unable to walk on his heels or toes. Whole exome sequence analysis identified a heterozygous nonmaternal variant of uncertain significance (c. 342 C>A, p. (S114R)) in the *KCNB1* gene—mitochondrial DNA analysis was normal. Current medications are hydroxyzine 10 mg at bedtime and trazodone 50 mg once daily. He is sociable and remains in a regular classroom 80% of the time.

The proband’s 6-year-old sister (patient B) was born at 36.5 weeks via C-section. Her Apgar scores were 9 at 1 min and 9 at 5 min. Her birth weight was 2.710 kg (10th percentile). The child was admitted to the neonatal intensive care unit due to respiratory distress of the newborn requiring one dose of surfactant with subsequent weaning off CPAP and supplemental O_2_ after 10 days. She experienced anemia of prematurity and feeding difficulties along with bilateral hyperflexion of hips. She passed newborn hearing screening with subsequent newborn metabolic screening also being normal and was discharged from the NICU at 18 days of life.

Patient B demonstrated delayed achievement of developmental milestones: at 12 months, she refused to walk or crawl, was unable to pull to sit/stand, and had persistent head lag. She rolled over at 13 months of age, sat unassisted at 15 months of age, and started crawling at 16–17 months of age. At 14 months, she demonstrated diffuse muscle hypotonia, lack of coordination, limited food acceptance, and slowed speech and language development only having three words at 20 months and being unable to follow simple commands. She passed an audiology exam at 12 months. At 17 months, she was diagnosed with a global development delay with no signs of regression.

At 17 months she was formally evaluated for hip dysplasia and found to have no abnormalities. The patient continued to have severe hypotonia; at 20 months, she was noted to have the muscular tone at the level of a 10-month-old. A swallowing study performed at this time for evaluation of dysphagia revealed mild deficits in oropharyngeal swallow due to reduced motor skills. A brain MRI performed at 19 months of age was normal.

At 23 months, patient B presented with behavioral challenges. Her mother described spells characterized by behavioral pause followed by screaming and shaking of the hands, frequent tantrums, staring spells accompanied by eye flutter, loss of head tone, and infrequent myoclonus without clonic, tonic, or clonic/tonic activity, all suggestive of absence seizures. Subsequently, at 2 years of age, she was diagnosed with a gait abnormality and was formally diagnosed with mild cerebral palsy in addition to autistic spectrum disorder. At 34 months of age an EEG was performed which failed to demonstrate ictal activity but showed abnormal mild, diffuse background slowing.

Patient B also demonstrated sleep difficulties, only sleeping for short periods throughout the night. Further, at 4 years of age patient B presented with complaints of intermittent severe muscle cramps in her legs. Muscle biopsy revealed no abnormalities. It was concluded that muscle cramping was not a myopathy, and likely had central nervous system origin. The patient’s guardian also reported drop attacks in which the patient would suddenly fall without warning. At 4 years and 7 months of age a Comprehensive Neuromuscular Disorders Panel (Invitae) covering 211 genes was ordered. A heterozygous pathogenic variant in ACADM was detected which when biallelically inherited causes medium-chain AcylCoA dehydrogenase deficiency. Repeat EEG revealed abnormal, bifrontal spike wave, consistent with stereotype in the setting of ASD.

Genetic evaluation at 5 years and 7 months of age was performed for the indications of DD/ID, ASD, and seizure disorder. No history of regressions or symptoms consistent with metabolic crises were noted. Morphometrics were normal with head circumference being at the 95th percentile. Dysmorphic features on physical examination included a prominent forehead with mild bossing, mild hypertelorism, wide and fleshy nose, low and wide columella, widely spaced large teeth, and difficulty walking with low tone the upper and lower extremities (Figure 1D).

Whole exome sequence analysis revealed the same, heterozygous nonmaternal variant of uncertain significance (c. 342 C>A), p. (S114R) in the *KCNB1* gene found in her older sibling Patient A. Patient B is currently taking clonidine 0.1 mg daily, hydroxyzine 10 mg at bedtime, lamotrigine 25 mg two tablets every 12 h, levetiracem 100 mg/mL, 3 mL every 12 h, and tizanidine 2 mg ½ tablet every 12 h.

In summary, patient A had a variant of unknown significance in a gene causing limb girdle muscular dystrophy identified on a neuromuscular panel done prior to exome sequencing. This variant was not verified on exome sequencing and the patient does not manifest symptoms consistent with this disease. Patient B had a heterozygous variant of unknown significance in a gene causing middle chain acetyl CoA dehydrogenase deficiency (MCAD) identified on a neuromuscular panel performed prior to exome sequencing. This disease manifests when homozygous/combined heterozygous variants are identified in a patient; her newborn screen was negative for increases in C8, C6, or C10. This variant was not verified on exome sequencing. Thus, the variant in *KCNB1* (Kv2.1) identified in these siblings via exome sequencing was the best explanation for their overlapping neurologic phenotype, and we pursued functional characterization of the *KCNB1* variant.

### Functional characterization of Kv2.1-S114R reveals slowing of activation and deactivation compared to wild-type Kv2.1

**Figure 2:**
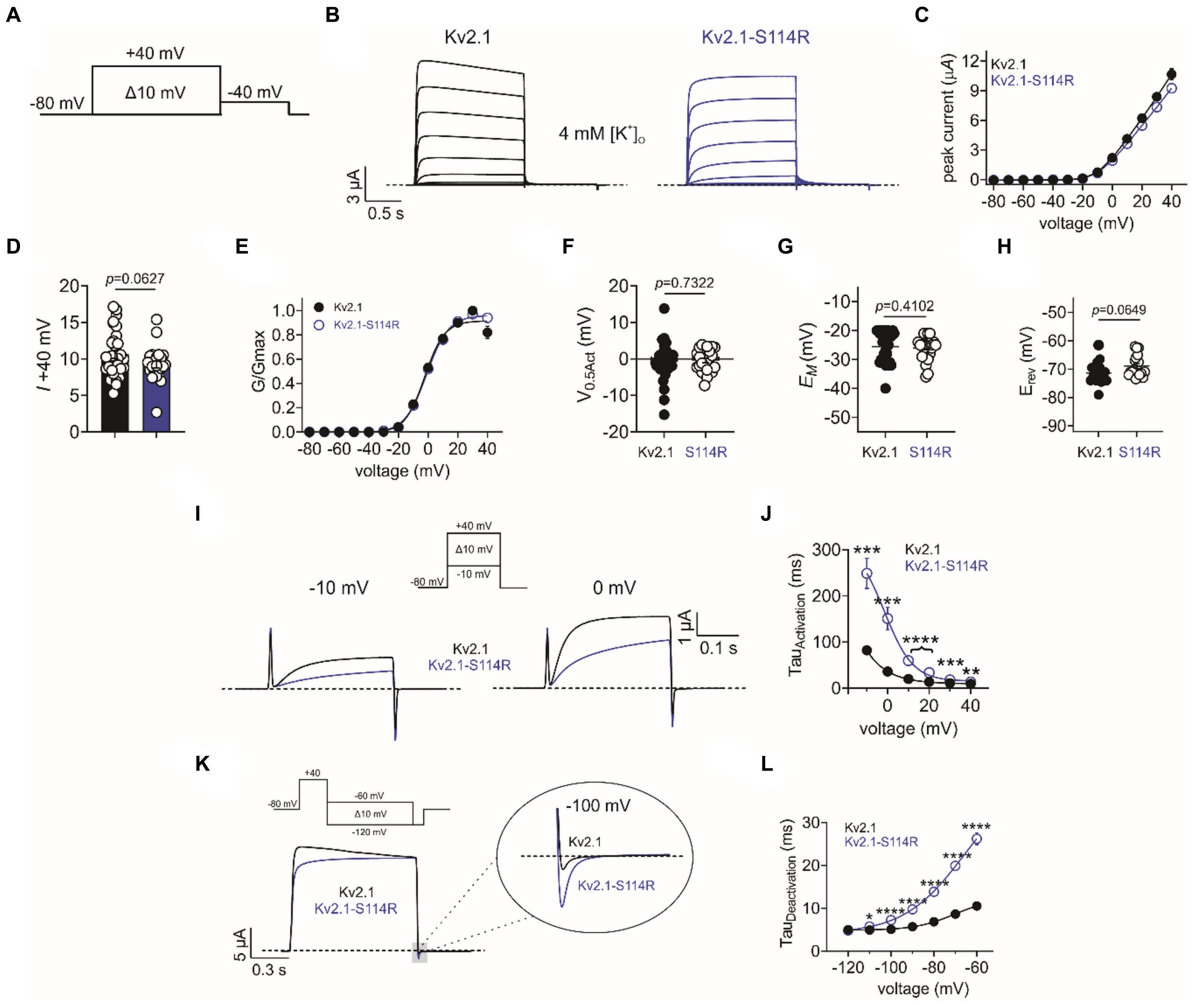
S114R slows Kv2.1 activation and deactivation. **(A)** Voltage protocol used to generate current–voltage relationships. **(B)** Mean traces recorded in response to the voltage protocol as in **A**, for wild-type (black) and S114R (blue) Kv2.1 channels expressed in *Xenopus* oocytes recorded in 4 mM [K^+^]_O_. Scale bar lower left inset (*n* = 24–31). **(C)** Mean I/V relationship for recordings as in **B** (*n* = 24–31). **(D)** Mean peak current density at +40 mV as in **B** (*n* = 24–31). **(E)** Mean normalized G/V relationship for recordings as in **B** (*n* = 24–31). **(F)** Mean *V*_0.5_ activation for recordings as in **E** (*n* = 24–31). **(G)** Mean unclamped oocyte membrane potential for Kv2.1 and Kv2.1-S114R expressing oocytes as in **B** (*n* = 24–31). **(H)** Mean *E*_REV_ for Kv2.1 and Kv2.1-S114R (*n* = 17–18). **(I)** Mean activation traces recorded in response to the voltage protocol, upper inset, for wild-type (black) and S114R (blue) Kv2.1 channels. Scale bars upper right inset (*n* = 12). **(J)** Mean activation rate versus voltage for Kv2.1 and Kv2.1-S114R as in **I** across the voltage range (*n* = 12). **(K)** Mean deactivation traces recorded in response to the voltage protocol; upper inset, for wild-type (black) and S114R (blue) Kv2.1 channels. Scale bars lower left inset (*n* = 12). **(L)** Mean activation rate versus voltage for Kv2.1 and Kv2.1-S114R as in **K** across the voltage range (*n* = 12).

Given the extensive neurological disruption associated with Kv2.1-S114R, we conducted cellular electrophysiological analysis to determine potential effects on channel function. At the time of writing, Kv2.1-S114R has not been previously reported as either a benign or pathogenic mutation and is not observed within large population cohorts (The Genome Aggregation Database; gnomAD). *Xenopus laevis* oocytes were injected with wild-type Kv2.1 (2 ng) or Kv2.1-S114R (2 ng) and incubated at 16°C for 24 h prior to recording with two-electrode voltage-clamp (TEVC). Both wild-type Kv2.1 and Kv2.1-S114R channels exhibited robust, outwardly rectifying voltage-dependent currents in response to a standard membrane depolarization “family” protocol (Figures 2A,B), with no difference observed in peak current magnitude between wild-type and mutant Kv2.1 across all voltage ranges tested (Figures 2C,D). Neither did S114R alter the voltage dependence of Kv2.1 activation (*V*_0.5Act_) (Figures 2E,F) or the mean resting membrane potential (*E*_M_) of unclamped oocytes (Figure 2G).

Next, we investigated whether S114R altered the potassium selectivity of Kv2.1, thereby shifting the reversal potential (*E*_REV_). Some previously characterized Kv2.1 mutants have been shown to alter ion selectivity and *E*_REV_ (Torkamani et al., 2014; Thiffault et al., 2015), while the N-terminal domain of, e.g., TREK-1 has been shown to play a pivotal role in ion selectivity (Veale et al., 2014). However, the S114R mutation had no effect on *E*_REV_, which was comparable to that of wild type (Figure 2H). Interestingly, S114R did alter Kv2.1 activation kinetics, slowing activation >threefold at some voltages (Figure 2I), with the greatest effects observed between −10 mV and 20 mV (Figure 2J). Similarly, S114R slowed deactivation between −110 and −60 mV, greater than twofold at some voltages (Figures 2K,L).

### S114R slows Kv2.1 gating processes

**Figure 3:**
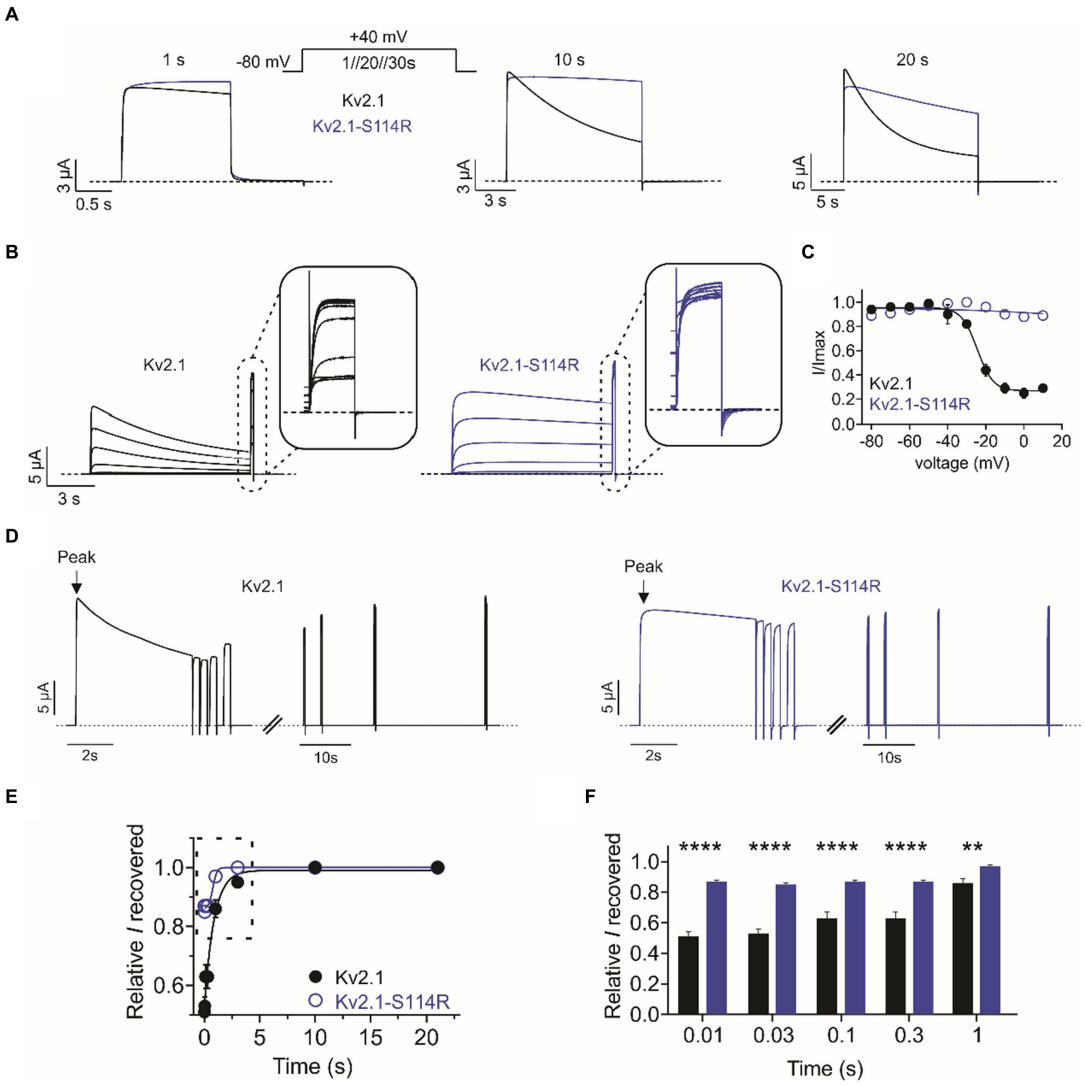
S114R greatly diminishes Kv2.1 inactivation. **(A)** Mean traces for wild-type (black) and S114R (blue) Kv2.1 channels, pulsed to +40 mV for 1 s, 10 s, and 20 s; voltage protocol, upper inset. Scale bars lower left inset (*n* = 10–31). **(B)** Mean traces for wild-type (black) and S114R (blue) Kv2.1 channels, expressed in oocytes measuring the fraction of non-inactivated channels in response to depolarizing voltage pulses. Scale bars lower left inset (*n* = 10). **(C)** Mean proportion of remaining non-inactivated current calculated from the circled portion of the traces as in **B** (*n* = 10). **(D)** Mean traces for wild-type (black) and S114R (blue) Kv2.1 channels, expressed in oocytes measuring residual current recovery. Arrows indicate peak current. Scale bars lower left inset (*n* = 12). **(E)** Residual current and recovery for wild-type (black) and S114R (blue) Kv2.1 channels for time points 0.01 s to 30 s versus peak current at +40 mV (*n* = 12). **(F)** Residual current and recovery for time points 0.01 s to 1 s as in **E** (*n* = 12).

Compared to wild-type, Kv2.1-S114R exhibited negligible steady-state inactivation across all voltages recorded from the I/V family (Figure 2B). Thus, we pursued this effect further by employing single-pulse protocols of +40 mV of increasing durations. At 1-, 10-, and 20-s pulse durations, wild type Kv2.1 exhibited characteristic slow steady-state inactivation. However, S114R showed no inactivation at 1- and 10-s at +40 mV (Figure 3A; left and middle), and only modest inactivation at 20 s (Figure 3A; right). To probe this further, we investigated whether S114R could alter the voltage-dependence of steady-state inactivation by using a protocol with 10 s depolarizing prepulses from −80 mV to +10 mV immediately followed by a +40 mV pulse (Figure 3B). The *V*_0.5_ of steady-state inactivation for wild-type Kv2.1 was −24 mV, which meant at a holding potential of, e.g., −20 mV, ~40% of channels were still available for activation. Strikingly, S114R exhibited no discernible inactivation across all holding potentials, meaning no channels entered the inactivated state and essentially all were available for activation (Figure 3C). Next, we measured the recovery from inactivation, which is an indicator of channel refractory period, by using a double-pulse protocol whereby consecutive +40 mV voltage pulses of 5 s are separated by increasing intervals from 0.01 to 30 s (Figure 3D). Compared to wild-type Kv2.1, S114R had an increased rate of recovery from inactivation between 0.01 and 3 s compared to wild-type, but their recovery rates were similar between 10 and 30 s (Figures 3E,F). The multiplex effects of the S114R variant on Kv2.1 channel function result in context-dependent effects on overall function, i.e., under some circumstances gain of function would be observed; under other circumstances, loss of function.

### Heterozygous mimic channel function

**Figure 4:**
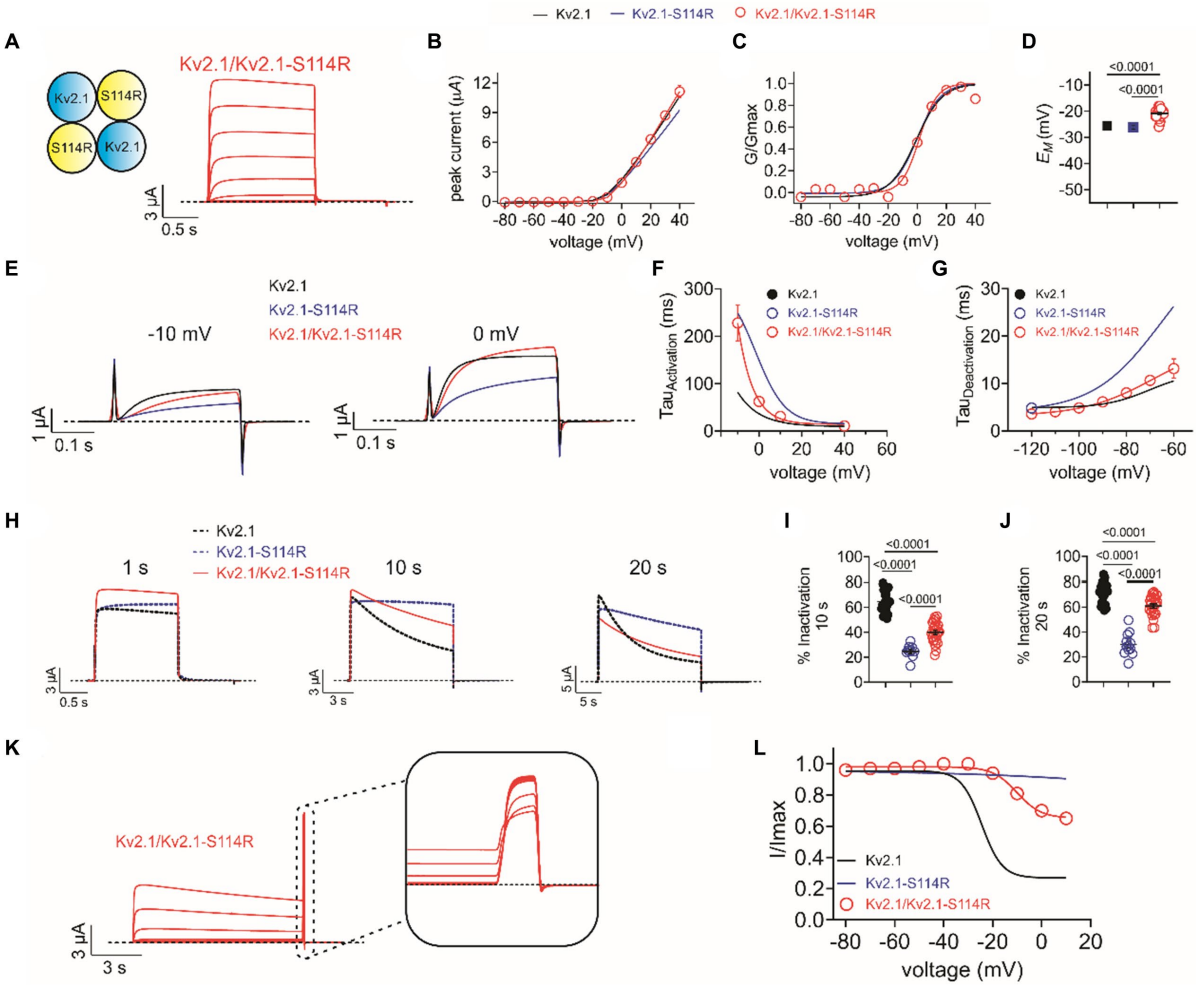
Heterozygous wild-type/S114R Kv2.1 channels exhibit intermediate gating kinetics. **(A)** Mean traces for homomeric, heterozygous Kv2.1 mutant Kv2.1-S114R channels (schematic, upper left inset) expressed in oocytes (*n* = 31). **(B)** Mean I/V relationship for recordings as in **A** (*n* = 31). **(C)** Mean normalized G/V relationship for recordings as in **A** (*n* = 31). **(D)** Mean unclamped oocyte membrane potential for Kv2.1 (black), Kv2.1-S114R (blue), and Kv2.1/Kv2.1-S114R (red) expressing oocytes as in **A** (*n* = 31). **(E)** Mean activation traces recorded in response to the voltage protocol, upper inset, for wild-type Kv1.2 (black), Kv1.2-S114R (blue), and Kv1.2/Kv1.2-S114R (red) channels. Scale bars lower left inset. Voltage protocol as in Figure 2I (*n* = 12). **(F)** Mean activation rate versus voltage for Kv2.1, Kv2.1-S114R, and Kv2.1/Kv2.1-S114R as in **E** across the voltage range (*n* = 12). **(G)** Mean deactivation rate versus voltage for wild-type Kv1.2 (black), Kv1.2-S114R (blue), and Kv1.2/Kv1.2-S114R (red) channels. Voltage protocol as in Figure 2K (*n* = 12). **(H)** Mean traces for wild-type Kv2.1 (black dashed line), Kv2.1-S114R (blue dashed line), and Kv2.1/Kv2.1-S114R (red) channels, pulsed to +40 mV for 1 s, 10 s, and 20 s. Voltage protocol as in Figure 3A (*n* = 25–31). **(I)** Mean percentage inactivation for 10 s at +40 mV for Kv2.1, Kv2.1-S114R, and Kv2.1/Kv2.1-S114R as in **H** (*n* = 12–27). **(J)** Mean percentage inactivation for 20 s at +40 mV for Kv2.1, Kv2.1-S114R, and Kv2.1/Kv2.1-S114R as in **H** (*n* = 12–27). **(K)** Mean trace for Kv2.1/Kv2.1-S114R, expressed in oocytes measuring the fraction of non-inactivated channels in response to depolarizing voltage pulses. Voltage protocol as in Figure 3B (*n* = 21). **(L)** Mean proportion of remaining non-inactivated current calculated from the circled portion of the trace as in **K**. For comparison Kv2.1 (black), Kv2.1-S114R (blue) as in Figure 3C (*n* = 21).

The above studies were conducted on homozygous mimicking all-wild type or all-S114R Kv2.1 channels to determine the mechanistic basis for altered channel activity in the mutant. However, patients A and B each had a single wild-type KCNB1 allele and a single mutant KCNB1 allele. Therefore, we compared the function of heterozygous-mimicking Kv2.1/Kv2.1-S114R channels by injecting oocytes 50/50 with wild-type and S114R Kv2.1 cRNA (Figure 4A). Compared to wild-type, heterozygous mutant channels had similar peak current magnitude and activation voltage dependence, although they appeared to result in moderately depolarized *E*_M_ (Figures 4B–D). Heterozygous channel activation rate was intermediate between that of homozygous mutant and wild-type, while heterozygous channel deactivation rate was closer to that of homozygous wild-type (Figures 4E–G). Heterozygous channel inactivation rate was intermediate between that of homozygous wild-type and mutant (Figures 4H–J), as was channel availability following inactivating depolarizing pulses (Figures 4K,L).

## Discussion

We report the discovery, clinical significance, and functional characterization of a novel pathogenic *KCNB1* mutation (p.S144R) in the cytoplasmic N-terminal region of Kv2.1. Prior to this study, 55 patients with *KCNB1* mutations had been previously studied. Clinical workups found 85% of the patients examined had developed epilepsy and all had developmental delays, with varying degrees of severity (de Kovel et al., 2017; Bar et al., 2020a; Xiong et al., 2022). Here, the siblings’ presentations, including dysmorphic features and developmental problems, were highly suggestive of an underlying genetic disorder. Genetic testing revealed that both patients possessed the same mutation—a heterozygous variant of previously uncertain significance (c. 342 C>A), p. (S114R) in the KCNB1 gene. Thus, the siblings were diagnosed with a *KCNB1*-related disorder. As indicated by Bar et al. (2020a), the siblings’ presentations are consistent with presentation of other known *KCNB1* patients. Aside from the nervous system, Kv2.1 is expressed in the GI tract, including pancreatic β-cells, where it regulates changes in cellular excitability, and insulin secretion, in response to glucose (MacDonald et al., 2002). In one study it was reported that a *KCNB1* SNP in the 3′ untranslated region (rs1051295) is associated with decreased insulin sensitivity, increased triglyceride and increased waist/hip ratio in the Chinese Han population, which can increase the risk for type 2 diabetes; this was not observed in the individuals in the current study (Zhang et al., 2013). *KCNB1* rs1051295 is also associated with risk of colon and rectal cancer, with an unknown mechanistic basis (Barbirou et al., 2020). We are not aware of *KCNB1* coding region variants associated with gastrointestinal tract disorders and none were noted in the clinical workups in the present study.

The absence of the variant in the patients’ mother and presence in both siblings suggests the biological father was an obligate *KCNB1* mutant carrier as the gene is inherited in a dominant manner, though gonadal mosaicism cannot be ruled out. The patients’ biological father was reported to have no health issues aside from learning disability, dyslexia and “autistic features.” As Uctepe et al. (2022) suggested, patients carrying a relatively mild *KCNB1*-related disease can pass on a more severe phenotype to their children. Interestingly, in the current case, both siblings presented a more severe phenotype than their biological father.

Patient B’s presentation was overall more severe than her brother’s. In addition to frequent tantrums, the child reports severe, persistent muscle cramps. It was concluded that these cramps are of central nervous system origin. The child is currently prescribed baclofen (5 mg) to reduce muscle cramping. Both patients are prescribed antiepileptics regularly. Patient A suffers from anxiety and depression and is treated accordingly. As mentioned, patient A has friends in school, performs well, and is in a regular classroom 80% of the time. Patient B is only 6 years old, and not yet in a regular classroom, so it is not yet possible to evaluate her performance and behavior in an academic setting.

There are limitations to this study. It is technically possible that the male patient’s symptoms are a consequence of hypoxia during the newborn period despite normal MRI. The female patient also carries a variant of undetermined significance in acyl-Coenzyme A dehydrogenase (ACADM). It is plausible that some of her symptoms are due to this variant existing in tandem with another undiagnosed variant in the same gene in trans versus the *KCNB1* mutation. Further, the mother also reported developmental delays and mental health concerns despite not carrying the *KCNB1* gene variant. Additionally, there is a maternal uncle with seizures indicating a possible confounding disorder in the children.

Given the complex genetic background, we functionally characterized the effects of the S114R substitution on Kv2.1 function. S114R markedly slowed the activation and deactivation and all but abolished steady-state inactivation at physiologically relevant durations. In the homozygous or heterozygous channel-mimicking conditions, S114R altered neither the peak Kv2.1 current nor the voltage-dependence of activation, but greatly slowed all channel gating processes. In addition, we utilized the reductionist oocyte expression system for this initial study, to evaluate the effects of the S114R variant on Kv2.1 ion conducting properties and gating kinetics. Oocytes are particularly well-suited for understanding the biophysical effects of ion channel variants in both the homozygous and heterozygous conditions, because each oocyte is injected with a precise amount of channel cRNA, the heterologously expressed currents are much larger than endogenous currents, and two-electrode voltage clamp recordings facilitate long recordings with challenging voltage protocols. Nevertheless, oocytes do not recapitulate the neuronal environment that is important for fully shaping Kv2.1 function in the brain. Future work could explore, for example, the potential effects on the non-conducting role of Kv2.1 in integrin-K^+^ channel complexes, considered important for normal neuronal migration, proliferation, survival and death (Forzisi and Sesti, 2022) and implicated in the abnormal neocortical development observed in *KCNB1* developmental epileptic encephalopathy (Bortolami et al., 2023).

Recently, another N-terminal *KCNB1* gain-of-function mutation, P17T, was shown to enhance currents as well as right-shift steady-state inactivation (Veale et al., 2022). The authors proposed that this right-shift in steady-state inactivation is in part the mechanism underlying the increase in current density, with more channels available for activation at depolarized voltages. S114R essentially abolishes steady-state inactivation in the homozygous condition, suggesting all channels should be available for activation; we observed no increase in current magnitude during a standard voltage family protocol, but we did observe higher sustained current during repetitive pulses in a voltage protocol designed to quantify the amount of channels available after inactivating pulses (Figure 3D).

Previously, it was shown that deleting the first 139 amino acids of the N-terminus slowed activation and deactivation kinetics Kv2.1 and abolished inactivation (VanDongen et al., 1990), highly consistent with what we observed for S114R and suggesting this residue is pivotal for all three gating types. Interestingly, the functional changes bought upon by the above referenced N-terminal truncation could be reversed by deleting an additional 318 residues from the C-terminal end, suggesting both domains are important for modulating Kv2.1 inactivation (VanDongen et al., 1990). Additionally, a regulatory domain (NRD) consisting of 59 amino acids (148–196) in the N-terminus of Kv2.1 was previously found to be important in gating. Replacement of the NRD in Kv2.1 with that of the same region in Kv2.3 slowed activation and deactivation and markedly slowed inactivation (Chiara et al., 1999). We did not see evidence for effects on Kv2.1 trafficking (there was no change in peak current magnitude) and accordingly, previous studies found a role for C-terminal, not N-terminal, motifs in Kv2.1 localization and trafficking (Lim et al., 2000; Jensen et al., 2017).

Kv2.1 is a major molecular correlate of the delayed rectifier potassium channels in cortical and hippocampal pyramidal neurons (Guan et al., 2007). Kv2.1 characteristic U-type inactivation is thought to be important during repetitive stimulation of neurons where it can dictate channel availability more so than P/C-type inactivation (Cheng et al., 2011). The effects of the S114R variant are complex. By slowing activation, it decreases current at the earliest time points following a depolarization but does not affect peak current across a longer depolarization (Figure 2I versus Figure 2C). By slowing deactivation, the variant increases tail currents observable at hyperpolarized potentials following a depolarization (Figure 2K). Finally, by dramatically slowing inactivation the S114R variant increases the peak current sustainable across a train of pulses because it reduces accumulation of channels in the inactivated state (Figures 3D–F). It is therefore difficult to categorize S114R as either a gain-of-function or loss-of-function variant; the effect is highly context dependent. Because of its multiplex effects on various components of Kv2.1 gating, it is difficult to predict whether inhibitors or openers would best treat the Kv2.1-S114R-associated condition. Other than genome editing or similar approaches to correct the actual mutation, small molecules that promote channel inactivation and/or activation would be desirable, as the heterozygous Kv2.1/Kv2.1-S114R channels show similar deactivation kinetics to wild-type Kv2.1, whereas activation and inactivation are much slower.

To our knowledge, this is the first study of its kind that compares the presentation of a *KCNB1* disorder in a sibling pair. The information gathered from this study could help to elucidate symptoms of *KCNB1* related disorder, aiding clinicians in diagnosing and treating *KCNB1* encephalopathy patients.

## Data availability statement

The original contributions presented in the study are included in the article/Supplementary material, further inquiries can be directed to the corresponding authors.

## Ethics statement

The studies involving humans were approved by University of Missouri Institutional Review Board. The studies were conducted in accordance with the local legislation and institutional requirements. The human samples used in this study were acquired from patients who were whole-exome sequenced as part of their clinical diagnosis. We received parent/guardian consent to study the identified gene variant and publish the findings. The University of Missouri Institutional Review Board reviewed and acknowledged this is a case report that is not considered human subject research. Written informed consent for participation was not required from the participants or the participants’ legal guardians/next of kin in accordance with the national legislation and institutional requirements. Written informed consent was obtained from the minor(s)’ legal guardian/next of kin for the publication of any potentially identifiable images or data included in this article.

## Author contributions

RM: Conceptualization, Formal analysis, Investigation, Methodology, Writing – original draft, Writing – review & editing. SB: Formal analysis, Investigation, Writing – review & editing. CI: Formal analysis, Investigation, Writing – review & editing. JK: Formal analysis, Investigation, Methodology, Writing – review & editing. RS: Conceptualization, Data curation, Methodology, Supervision, Writing – original draft, Writing – review & editing. GA: Conceptualization, Funding acquisition, Methodology, Supervision, Writing – original draft, Writing – review & editing.
