# Supplementary material for: A novel autism-associated KCNB1 mutation dramatically slows Kv2.1 potassium channel activation, deactivation and inactivation
Source: Front Cell Neurosci. 2024 Jul 29;18:1438101. doi: 10.3389/fncel.2024.1438101 (PMC11317242; doi:10.3389/fncel.2024.1438101)
Supplement: Supplementary file 1 [file Table_1.DOCX]

|  | **Peak current at +40 (µA)** | **V_0.5_ Normalized tail current (mV)** | **Slope (mV)** | ***E_M_* (mV)** | ***E_rev_* (mV)** |
| --- | --- | --- | --- | --- | --- |
| **Kv2.1** | 10.65 ± 0.56 (*n*=31) | -0.70 ± 0.75 (*n*=31) | 8.43 ± 0.55 (*n*=31) | -25.51 ± 0.95 (*n*=31) | -71.35 ± 0.95 (*n*=17) |
| **Kv2.1-S114R** | 9.25 ± 0.48 (*p*=0.0627; *n*=24) | 0.13 ± 0.64 (*p*=0.7322; *n*=24) | 7.65 ± 0.43 (*p*=0.2631; *n*=24) | -26.08 ± 0.80 (*p*=0.4102; *n*=24) | -68.86 ± 0.90 (*p*=0.0649; *n*=18) |

**Table 1.** Biophysical properties of homozygous channels. Statistics versus Kv2.1 wild type. Values indicate mean ± SEM. Statistical comparisons by t-test.

| **Tau activation (ms)** | **Kv2.1** | **Kv2.1-S114R** |
| --- | --- | --- |
| -10 mV | 82.16 ± 9.29 (*n*=12) | 248.95 ± 32.71 (*p*=0.0003; *n*=12) |
| 0 mV | 36.05 ± 2.88 (*n*=12) | 150.85 ± 24.43 (*p*=0.0006; *n*=12) |
| 10 mV | 19.86 ± 1.42 (*n*=12) | 59.67 ± 6.28 (<0.0001; *n*=12) |
| 20 mV | 13.68 ± 0.96 (*n*=12) | 33.51 ± 3.21 (<0.0001; *n*=12) |
| 30 mV | 10.78 ± 0.81 (*n*=12) | 17.77 ± 1.53 (*p*=0.0009; *n*=12) |
| 40 mV | 9.43 ± 0.71 (*n*=12) | 13.44 ± 1.04 (*p*=0.0047; *n*=12) |

**Table 2.** Time constant of activation of homozygous channels. Statistics versus Kv2.1 wild type. Values indicate mean ± SEM. Statistical comparisons by t-test.

| **Tau deactivation (ms)** | **Kv2.1** | **Kv2.1-S114R** |
| --- | --- | --- |
| -120 mV | 5.01 ± 0.17 (*n*=12) | 4.83 ± 0.24 (*p*=0.5453; *n*=12) |
| -110 mV | 4.94 ± 0.11 (*n*=12) | 5.77 ± 0.26 (*p*=0.0107; *n*=12) |
| -100 mV | 5.12 ± 0.12 (*n*=12) | 7.27 ± 0.31 (<0.0001; *n*=12) |
| -90 mV | 5.70 ± 0.12 (*n*=12) | 9.77 ± 0.44 (<0.0001; *n*=12) |
| -80 mV | 6.86 ± 0.20 (*n*=12) | 13.87 ± 0.68 (<0.0001; *n*=12) |
| -70 mV | 8.67 ± 0.29 (*n*=12) | 19.92 ± 1.02 (<0.0001; *n*=12) |
| -60 mV | 10.54 ± 0.81 (*n*=12) | 26.23 ± 1.38 (<0.0001; *n*=12) |

**Table 3**. Time constant of deactivation of homozygous channels. Statistics versus Kv2.1 wild type. Values indicate mean ± SEM. Statistical comparisons by t-test.

|  | **Peak current at +40 (mV)** | **V_0.5_ Normalized tail current (mV)** | **Slope (mV)** | ***E_M_* (mV)** |
| --- | --- | --- | --- | --- |
| **Kv2.1/Kv2.1-S114R** | 11.31 ± 0.60 (*n*=31) | 1.05 ± 0.62 (*n*=31) | 6.16 ± 0.27 (*n*=31) | -20.83 ± 0.33 (*n*=31) |

**Table 4.** Biophysical properties of Kv2.1/Kv2.1-S114R heterozygous channels. Values indicate mean ± SEM.

| **Tau activation (ms)** | **Kv2.1/Kv2.1-S114R** |
| --- | --- |
| -10 mV | 228.23 ± 38.15 (*n*=12) |
| 0 mV | 62.40 ± 5.90 (*n*=12) |
| 10 mV | 30.79 ± 2.30 (*n*=12) |
| 20 mV | 19.26 ± 1.40 (*n*=12) |
| 30 mV | 13.71 ± 1.04 (*n*=12) |
| 40 mV | 10.78 ± 0.85 (*n*=12) |

**Table 5.** Time constant of activation of Kv2.1/Kv2.1-S114R heterozygous channels. Values indicate mean ± SEM.

| **Tau deactivation (ms)** | **Kv2.1/Kv2.1-S114R** |
| --- | --- |
| -120 mV | 3.59 ± 0.43 (*n*=12) |
| -110 mV | 4.07 ± 0.41 (*n*=12) |
| -100 mV | 4.86 ± 0.44 (*n*=12) |
| -90 mV | 6.13 ± 0.53 (*n*=12) |
| -80 mV | 8.03 ± 0.70 (*n*=12) |
| -70 mV | 10.66 ± 1.02 (*n*=12) |
| -60 mV | 13.15 ± 2.03 (*n*=12) |

**Table 6.** Time constant of deactivation of Kv2.1/Kv2.1-S114R heterozygous channels. Values indicate mean ± SEM.

| **% Inactivation at +40 mV** | **Kv2.1** | **Kv2.1-S114R** | **Kv2.1/Kv2.1-S114R** |
| --- | --- | --- | --- |
| 10 Seconds | 64.61 ± 1.70 (*n*=25) | 24.65 ± 1.64 (*n*=10) | 39.93 ± 1.67 (*n*=25) |
| 20 Seconds | 71.61 ± 1.49 (*n*=27) | 30.04 ± 2.64 (*n*=12) | 60.98 ± 1.65 (*n*=25) |

**Table 7.** Percentage inactivation at +40 mV for homozygous and heterozygous channels. Values indicate mean ± SEM.

**We have used Dryad as a repository for all of the raw data used to generate Figures 2-4 of this manuscript. The repository can be accessed via the following link:**

**doi:10.5061/dryad.6hdr7sr8t**
